# Tandem Duplication and Random Loss for mitogenome rearrangement in *Symphurus* (Teleost: Pleuronectiformes)

**DOI:** 10.1186/s12864-015-1581-6

**Published:** 2015-05-06

**Authors:** Wei Shi, Li Gong, Shu-Ying Wang, Xian-Guang Miao, Xiao-Yu Kong

**Affiliations:** CAS Key Laboratory of Tropical Marine Bio-resources and Ecology, South China Sea Institute of Oceanology, Chinese Academy of Sciences, 164 West Xingang Road, Guangzhou, 510301 PR China

**Keywords:** Flatfish, Mitogenome, Gene rearrangement, O_L_-like structure, Mitochondrial replication

## Abstract

**Background:**

The mitochondrial genomes (mitogenomes) of flatfishes (Pleuronectiformes) exhibit highly diversified types of large-scale gene rearrangements. We have reported that the mitogenomes of *Crossorhombus azureus* (Bothidae), *Samariscus latus* (Samaridae) and *Cynoglossus* fishes (Cynoglossidae) show different types of gene rearrangements.

**Results:**

In the present study, the complete mitogenomes of two *Symphurus* species (Cynoglossidae), *Symphurus plagiusa* and *Symphurus orientalis*, were determined. The gene order in the *S. plagiusa* mitogenome is the same as that of a typical vertebrate (without any gene rearrangements). Surprisingly, large-scale gene rearrangements have occurred in *S. orientalis*. In the rearranged fragment from the control region (CR) to the WANCY tRNA cluster (tRNA cluster of *tRNA-W*, *tRNA-A*, *tRNA-N*, *tRNA-C* and *tRNA-Y*) in the *S. orientalis* mitogenome, *tRNA-V* and *tRNA-M* have been translocated to the 3’ end of the *16S rRNA* gene, with six large intergenic spacers over 20 bp in length. In addition, an origin for light-strand replication (O_L_) structure that is typically located in the WANCY region was absent in both the *S. plagiusa* and *S. orientalis* mitogenomes. It is generally recognized that a sequence in the WANCY region that encodes tRNAs forms a hairpin structure (O_L_-like structure) and can act as the O_L_ when the typical locus is lost. Moreover, an additional O_L_-like structure was identified near the control region in the *S. plagiusa* mitogenome.

**Conclusions:**

The positions of the intergenic spacers and the rearranged genes of the *S. orientalis* mitogenome strongly indicate that the mechanism underlying the rearrangement of this mitogenome was Tandem Duplication and Random Loss. Additionally, two O_L_-like regions substituting for the typical locus were found in the *S. plagiusa* mitogenome. We speculate that the ancestral mitogenomes of *S. plagiusa* and *S. orientalis* also had this characteristic, such that if both O_L_-like structures functioned during mitochondrial replication, they could initiate duplicate replications of the light strand (L-strand), leading to duplication of the region between the two structures. We consider that this mechanism may account for the gene duplication that occurred during the gene rearrangement process in the evolution of the ancestral mitogenome to the *S. orientalis* mitogenome.

**Electronic supplementary material:**

The online version of this article (doi:10.1186/s12864-015-1581-6) contains supplementary material, which is available to authorized users.

## Background

Vertebrate mitochondrial genomes (mitogenomes) typically contain the same 37 genes [1]. The order of these genes is generally considered conservative in most vertebrate genomes; however, gene rearrangements also have been found in many taxa, such as birds [2-4], reptiles [5,6], amphibians [7,8], and fishes [9-11]. Teleosts, with the largest number of published complete mitogenome sequences, show only a few gene rearrangement events [9-13]. In most cases, a teleostean group has only one type or a set of similar gene rearrangements [9-14]. However, the flatfish (Pleuronectiformes) mitogenomes exhibit the most diversified types of large-scale gene rearrangements. In the mitogenomes of *Cynoglossus* fishes (tongue soles, Cynoglossidae), the control region is translocated, and a tRNA (transfer ribonucleic acid) gene is inverted [10]. In contrast, no gene rearrangements have been found in soles (Soleidae), the closest family to Cynoglossidae fishes [15-17]. The mitogenome of *Crossorhombus azureus* (Bothidae) contains genomic-scale gene rearrangements characterized by the protein-coding gene *ND6* and seven tRNA genes encoded on the light strand (L-strand; H- versus L-strands are defined by studies on AT- and GC-skewing [18,19]) that are clustered together [20]. A third type of gene rearrangement was detected in the *Samariscus latus* (Samaridae) mitogenome [21]. Distinct from the above-mentioned flatfishes, the gene rearrangement in this species is characterized by the duplication and translocation of the control region (CR); simultaneously, the genes located between the two CRs are divided into two clusters in which their relative gene orders have been maintained [21].

Several models have been proposed to explain gene rearrangements in animal mitogenomes. The Recombination model involves the breakage and rejoining of participating DNA strands [22]. The Tandem Duplication and Random Loss (TDRL) model posits that rearrangements of mitochondrial gene order occurred via tandem duplications of certain genes followed by random deletion of some of the duplications [23,24]. Two additional hypotheses are described in the Tandem Duplication and Non-random Loss (TDNL) [25] and tRNA mis-priming models [26,27]. For the gene rearrangements in flatfishes, none of the models mentioned above can provide a perfect explanation. Thus, Kong et al. [10] developed a model of inverse duplication and deletion of redundant genes to explain the gene rearrangements in tongue soles. Subsequently, Shi et al. [20] proposed the Dimer-Mitogenome and Non-Random Loss model (DMNR), which inferred the course of gene rearrangements in *C. azureus*. Recently, for the rearrangement events in the *S. latus* mitogenome, Shi et al. [21] proposed the Double Replications and Random Loss model.

Of these mechanisms proposed to explain mitochondrial gene rearrangements, the TDRL model is generally considered the most popular and important in vertebrates [9,23,24,28,29]. Generally, when decrypting gene rearrangements with the TDRL model, it is always necessary to propose multiple duplication and loss steps. It is therefore difficult to trace which steps preserved the functional genes and which DNA segments degenerated to pseudogenes or intergenic spacers. In other words, large-scale gene rearrangements cannot readily yield integrated evidence for the TDRL model [9,23,24,28,29]. San Mauro et al. [29] also indicated that the evidence for this model in the form of duplicated genes that either remain functional or have become pseudogenes in the process of being eliminated is rather limited.

In the present study, the complete mitogenomes of two flatfishes, *Symphurus plagiusa* and *Symphurus orientalis*, were sequenced. Surprisingly, the gene order of the *S. plagiusa* mitogenome resembles that of a typical vertebrate (un-rearranged gene order), whereas that of *S. orientalis* shows large-scale gene rearrangements. This is the first report of mitogenomes with a typical gene order and large-scale gene rearrangements within the same teleost genus. The characteristics of the gene order and intergenic spacers in *S. orientalis* provide clear evidence for the TDRL model, accounting for the gene rearrangements in the *S. orientalis* mitogenome*.*

## Methods

### Ethics statement

Ethical approval was not required for the present study because the examined specimens were commonly captured marine economic fishes, and all of the fish specimens were already dead when we obtained them and were sourced from commercial fisheries. Additionally, these species were not included in the IUCN list of endangered species (http://www.iucnredlist.org).

### Sampling, DNA extraction, PCR and sequencing

Specimens of *S. plagiusa* and *S. orientalis* were collected from Tampa Bay, Florida (USA) and Taiwan (China), respectively. A portion of the epaxial musculature was excised from fresh specimens and immediately stored at −70°C. Total genomic DNA was extracted using the SQ Tissue DNA Kit (OMEGA) following the manufacturer’s protocol. Based on alignments and comparisons of complete mitochondrial sequences from flatfishes, dozens of primer pairs were designed for amplification of the mtDNA genomes (Additional file 1: Table S1 and Additional file 2: Table S2). More than 30 bp of overlapping fragments between tandem regions were used to ensure correct assembly and integrity of the complete sequence.

PCR (polymerase chain reaction) was performed in a 25 μl reaction volume containing 2.0 mM MgCl_2_, 0.4 mM of each dNTP, 0.5 μM of each primer, 1.0 U Taq polymerase (Takara, China), 2.5 μl of 10x Taq buffer, and approximately 50 ng of DNA template. PCR cycling conditions included an initial denaturation at 95°C for 3 min, followed by 30–35 cycles of denaturation at 94°C for 45 s, annealing at 45–55°C for 45 s, and elongation at 68–72°C for 1.5–5 min. The PCR reactions were completed by a final extension at 72°C for 5 min. The PCR products were purified with the Takara Agarose Gel DNA Purification Kit (Takara, China) and used directly as templates for cycle sequencing reactions. Sequence-specific primers were further designed and used as walking primers for both strands of each fragment on an ABI 3730 DNA sequencer (Applied Biosystems, USA). The mtDNA sequences of *S. plagiusa* and *S. orientalis* have been submitted to GenBank under the accession numbers JQ639061 and KP992899, respectively.

### Sequence analysis

Sequenced fragments were assembled to create complete mitochondrial genomes using CodonCode Aligner v3 and BioEdit v7 [30]. During the processing of large fragments and walking sequences, regular manual examinations were performed to ensure reliable assembly of the genome sequence. Annotation and boundary determination of protein-coding and rRNA (Ribosomal ribonucleic acid) genes were performed using NCBI-BLAST (http://blast.ncbi.nlm.nih.gov). TRNA genes and their secondary structures were identified using tRNAscan-SE 1.21 [31], setting the cut-off values to 1 when necessary.

## Results

### Features of the genomes

The complete mitogenomes of *S. plagiusa* and *S. orientalis* were 17040 bp and 17498 bp in length, respectively, and contained 13 protein-coding genes, 22 tRNA genes, and 2 rRNA genes as well as one CR. Most of these genes were encoded by the heavy strand (H-strand), except *ND6* and eight tRNA genes, which were encoded on the L-strand (Table 1). The 22 tRNA genes were interspersed among rRNAs and protein-coding genes, and all tRNAs can be folded into typical cloverleaf structures. The location of the CR was between the *tRNA-P* and *tRNA-F* genes, as is typical for teleosts. Compared with the CR sequences of the other flatfishes, the symbolic structures of the two *Symphurus* CRs were present as in other bony fishes [16,20,21,32,33]. The typical origin for L-strand replication (O_L_), which is usually located inside the WANCY cluster (tRNA cluster of *tRNA-W*, *tRNA-A*, *tRNA-N*, *tRNA-C* and *tRNA-Y*), was not found in either of the two *Symphurus* mitogenomes at this location.

**Table 1:** **Features of the mitogenomes of Symphurus plagiusa (Left) and Symphurus orientalis (Right)**

**Gene**	**Position**		**Length (bp)**	Intergenic region*	**Strand**	**Gene**	**Position**		**Length (bp)**	**Intergenic region**	**Strand**
	**From**	**To**					**From**	**To**			
*tRNA-Phe* (*F*)	1	69	69	0	H	*tRNA-Phe*	1	71	71	0	H
*12S*	70	1016	947	0	H	*12S*	72	1020	949	65	H
*tRNA-Val* (*V*)	1017	1086	70	0	H	*16S*	1086	2796	1711	98	H
*16S*	1087	2793	1707	0	H	*tRNA-Met*	2895	2964	70	100	H
*tRNA-Leu* ^*UUA*^ (*L1*)	2794	2866	73	0	H	*tRNA-Val*	3065	3136	72	31	H
*ND1*	2867	3841	975	2	H	*tRNA-Leu* ^*UUA*^	3168	3240	73	0	H
*tRNA-Ile* (*I*)	3844	3913	70	-2	H	*ND1*	3241	4212	972	7	H
*tRNA-Gln* (*Q*)	3912	3982	71	-1	L	*tRNA-Ile*	4220	4289	70	-2	H
*tRNA-Met* (*M*)	3982	4050	69	124	H	*tRNA-Gln*	4288	4358	71	117	L
*ND2*	4175	5227	1053	42	H	*ND2*	4476	5522	1047	37	H
*tRNA-Trp* (*W*)	5270	5339	70	0	H	*tRNA-Trp*	5560	5628	69	0	H
*tRNA-Ala* (*A*)	5340	5408	69	0	L	*tRNA-Ala*	5629	5697	69	0	L
*tRNA-Asn* (*N*)	5409	5479	71	2	L	*tRNA-Asn*	5698	5770	73	3	L
*tRNA-Cys* (*C*)	5482	5546	65	3	L	*tRNA-Cys*	5774	5839	66	4	L
*tRNA-Tyr* (*Y*)	5550	5616	67	1	L	*tRNA-Tyr*	5844	5910	67	10	L
*COI*	5618	7168	1551	0	H	*COI*	5921	7471	1551	0	H
*tRNA-Ser* ^*UCA*^ (*S1*)	7169	7239	71	19	L	*tRNA-Ser* ^*UCA*^	7472	7542	71	19	L
*tRNA-Asp* (*D*)	7259	7327	69	4	H	*tRNA-Asp*	7562	7630	69	5	H
*COII*	7332	7925	594	96	H	*COII*	7636	8326	691	0	H
*tRNA-Lys* (*K*)	8022	8097	76	1	H	*tRNA-Lys*	8327	8400	74	2	H
*ATP8*	8099	8266	168	-10	H	*ATP8*	8403	8570	168	-10	H
*ATP6*	8257	8940	684	2	H	*ATP6*	8561	9244	684	-1	H
*COIII*	8943	9731	789	65	H	*COIII*	9244	10029	786	-1	H
*tRNA-Gly* (G)	9797	9864	68	0	H	*tRNA-Gly*	10029	10096	68	0	H
*ND3*	9865	10215	351	-2	H	*ND3*	10097	10447	351	-2	H
*tRNA-Arg* (*R*)	10214	10282	69	0	H	*tRNA-Arg*	10446	10514	69	0	H
*ND4L*	10283	10579	297	-7	H	*ND4L*	10515	10811	297	-7	H
*ND4*	10573	11953	1381	0	H	*ND4*	10805	12184	1380	1	H
*tRNA-His* (*H*)	11954	12022	69	0	H	*tRNA-His*	12186	12253	68	0	H
*tRNA-Ser* ^*UGC*^ (*S2*)	12023	12090	68	2	H	*tRNA-Ser* ^*UGC*^	12254	12321	68	2	H
*tRNA-Leu* ^*GUA*^ (*L2*)	12093	12164	72	0	H	*tRNA-Leu* ^*GUA*^	12324	12397	74	0	H
*ND5*	12165	13985	1821	-4	H	*ND5*	12398	14233	1836	4	H
*ND6*	13982	14500	519	0	L	*ND6*	14238	14756	519	0	L
*tRNA-Glu* (*E*)	14501	14569	69	2	L	*tRNA-Glu*	14757	14825	69	3	L
*Cytb*	14572	15711	1140	1	H	*Cytb*	14829	15969	1141	0	H
*tRNA-Thr* (*T*)	15713	15781	69	-1	H	*tRNA-Thr*	15970	16042	73	-1	H
*tRNA-Pro* (*P*)	15781	15849	69	0	L	*tRNA-Pro*	16042	16110	69	0	L
O_L_-like Seq.	16983	17034	52	/	L	D-loop	16111	17498	1388	0	H
D-loop	15850	17040	1191	0	H						

*Intergenic region: non-coding bases between the feature on the same line and the line below, with a negative number indicating an overlap.

### Two different mitochondrial gene orders in the genus *Symphurus*

The gene order of the *S. plagiusa* mitogenome is the same as that of a typical vertebrate, while that of *S. orientalis* contains large-scale gene rearrangements. This phenomenon is rare in vertebrates and the first report in teleosts. The difference between the two mitogenomes lies between the CR and the WANCY region. The gene order of this region in the *S. plagiusa* mitogenome is CR*-F-12S-V-16S-L1-ND1-I-Q-M-ND2-WANCY*, which is the same as that of a typical vertebrate, while this region has been rearranged to CR-*F-12S-16S-M-V-L1-ND1-I-Q-ND2-WANCY* in the *S. orientalis* mitogenome.

In the rearranged fragment spanning the region from the CR to the WANCY region in the *S. orientalis* mitogenome, six large intergenic spacers greater than 20 bp in length remain: Gap A, between *12S* and *16S* (65 bp); Gap B, between *16S* and *tRNA-M* (98 bp); Gap C, between *tRNA-M* and *tRNA-V* (100 bp); Gap D, between *tRNA-V* and *tRNA-L1* (31 bp); Gap E, between *tRNA-Q* and *ND2* (117 bp); and Gap F, between *ND2* and *tRNA-W* (37 bp).

## Discussion

### Which mechanisms account for the gene rearrangements in *S. orientalis* mitogenome?

To date, approximately 1,500 complete mtDNA sequences have been determined in teleosts, and several types of gene rearrangements have been reported [17,33-36]. In the taxa showing gene rearrangements, species across the entire taxon typically show the same or similar rearrangement events [4,6,14]. That is, at the intra-family or intra-genus level, few groups include species with the typical (unchanged) gene order together with species with large-scale gene rearrangements. Nevertheless, this unique phenomenon occurs in the flatfish genus *Symphurus*, as described above.

Of the models that have been proposed to explain gene rearrangements in animal mitogenomes, which model most likely applies to the *S. orientalis* mitogenome? The recombination model is only suitable for block interchanges of small fragments, and this model is quite rare in the mitochondrial genome. As for the tRNA mis-priming [26,27] and TDNL models [25], there are no obvious corresponding model rules in the *S. orientalis* mitogenome.

Several species within three flatfish groups (Bothidae, Samaridae and Cynoglossidae) have been reported to possess different types of gene rearrangements. Among them, *Cynoglossus* fishes belong to Cynoglossidae, the same family as *Symphurus.* However, the rearrangements in the *Cynoglossus* mitogenomes are characterized by inverted tRNA genes, which were not present in the *S. orientalis* mitogenome. The rearrangement characteristics of the other flatfish groups also differ from those of *S. orientalis*.

We numbered the gene order of the *S. plagiusa* mitogenome (typical gene order) from CR to WANCY in the following series: CR*–F* (1)*–12S* (2)*–V* (3)*–16S* (4)*–L1* (5)*–ND1* (6)*–I* (7)*–Q* (8)*–M* (9)*–ND2* (10)*–W* (11) *–A* (12) *–N* (13) *CY*. Following this scheme, the corresponding sequence of *S. orientalis* would be CR–*F* (1)*–12S* (2)*–16S* (4)*–M* (9)*–V* (3)*–L1* (5)*–ND1* (6)*–I*(7)*–Q* (8)*–ND2* (10)*–W* (11) *–A* (12) *–N* (13) *CY*. Based on this numeric order, the genes from *F* to *N* (1–13) in *S. orientalis* can be divided into two gene clusters: 1–2–(4–9) and (3–5–6–7–8)–10–11–12–13, each of which retains the conserved relative gene order, from low to high. It is reasonable to assume that the two clusters were derived from a tandem-duplicated DNA fragment that spanned genes from at least *V* (3) to *M* (9) in the typical gene order. Thereafter, one of each pair of duplicated genes was randomly lost: 1– 2 –(3 –4 –5 –6 –7 –8 –9) (3 –4 –5 –6 –7 –8 –9)–10 –11 –12 –13. Within the scope of our current knowledge, this rearrangement process represents the most parsimonious and reasonable hypothesis.

### The process of gene duplication

How did the duplication occur? Moreover, was the fragment from *V* (3) to *M* (9) or a longer fragment duplicated? Many studies have reported similar duplications. For example, Fujita et al. [37] found that several lineages of parthenogenetic lizards harbor large, tandem duplications, which are hypothesized to represent intermediate stages in gene rearrangement. These authors suggested that the slipped-strand mispairing mechanism could have been responsible for generating the duplications in the mitogenomes of these lizards.

**Figure 1:**
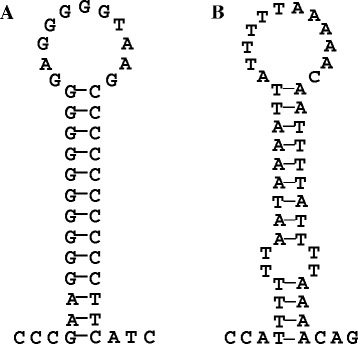
Typical teleostean O_L_ structure and O_L_-like structure in the *Symphurus plagiusa* mitogenome. **(A)** Stem-loop structure of the O_L_ in the *Cynoglossus semilaevis* mitogenome; **(B)** the O_L_-like structure in the *Symphurus plagiusa* mitogenome.

A special phenomenon observed in the *Symphurus* mitogenomes indicates that the underlying mechanism is different from slipped-strand mispairing and can help us to explain this duplication process. Generally, there are only two stable non-coding regions in vertebrate mitogenomes: the CR and an approximately 40 nucleotide-long segment containing the origin for L-strand replication (O_L_), which is usually located inside the WANCY cluster at approximately two thirds of the genomic distance away from the CR. At O_L_, the parental H-strand is displaced as a single strand by the nascent H-strand and adopts a stable hairpin structure (Figure 1A) that serves as the initiation site for L-strand DNA synthesis. We searched all of the complete mitogenome sequences of flatfishes in the GenBank database and found that most contained conserved O_L_ sequences, including *Cynoglossus* fishes (Figure 1A), which are in the same family as the *Symphurus* species and also show gene rearrangements. However, neither of the two *Symphurus* fish mitogenomes contains a typical O_L_ region in the WANCY cluster.

O_L_ sequence loss has been reported in some other vertebrate mitogenomes, and it was suggested that a sequence in the WANCY region that encodes a tRNA forms a hairpin structure (O_L_-like structure) and acts as the O_L_ [38-40]. Seligmann and Labra [41] tested whether a natural absence of an O_L_ is associated with a greater capacity for the formation of O_L_-like structures by WANCY tRNA genes in lepidosaurian taxa. These authors concluded that WANCY tRNA genes form more O_L_-like structures in the absence of a regular O_L_ than in its presence.

More interestingly, in the *S. plagiusa* mitogenome, within the CR and very close to *tRNA-F*, we found a hairpin structure that is very similar to the typical O_L_ of vertebrate mitogenomes (Table 1 and Figure 1B). This finding means that while no typical O_L_ region is present in the *S. plagiusa* mitogenome, there are two regions with the potential ability to form O_L_-like structures and initiate the replication of the L-strand. The existence of multiple O_L_s in vertebrate mitochondria has been demonstrated in many studies [42-44]. These features of multiple replication origins most likely also appeared in the mitogenome of the common ancestor of *S. plagiusa and S. orientalis* and were inherited by *S. plagiusa* due to its stable mitogenome structure. In contrast, these features would have been lost in the *S. orientalis* mitogenome because of the large-scale gene rearrangements. Therefore, in the ancestral mitogenome with the aforementioned dual O_L_-like structures, if only one is functioning, mitochondrial replication would occur normally, but if both structures functioned at the same time, the region between the two structures could be duplicated.

**Figure 2:**
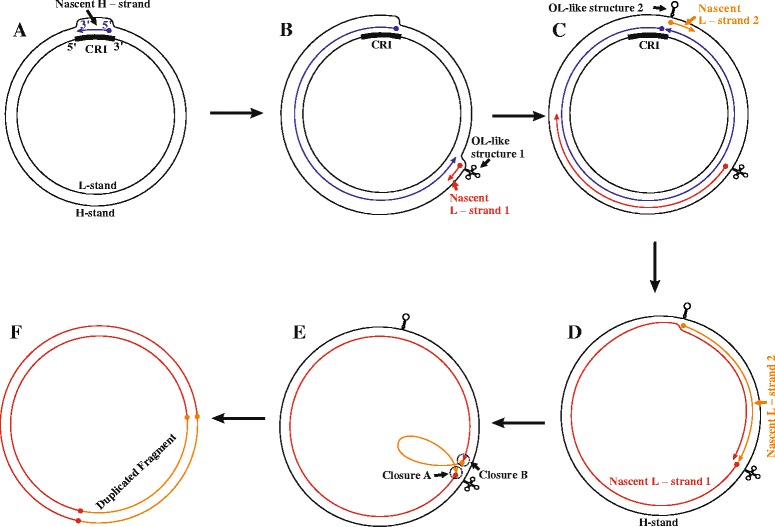
A duplication between the CR and the *N* gene caused by two O_L_-like structures. **(A)** H-strand synthesis is initiated at an origin for light-strand replication (O_H_). **(B)** L-strand synthesis is initiated at O_L_-like structure 1 when the replication fork reaches approximately two thirds of the genomic distance from the CR. **(C)** H-strand synthesis is terminated, and O_L_-like structure 2 initiates a second round of L-strand synthesis. **(D)** Both L-strand synthesis events are terminated at the WANCY region. **(E)** Connection of the 5’ end of Nascent L-strand 1 to the 3’ end of Nascent L-strand 2 and of the 3’ end of Nascent L-strand 1 to the 5’ end of Nascent L-strand 2. **(F)** The genes between the CR and *N* are, thus, duplicated.

Accordingly, we speculate that the two ends of the duplicated fragment fall in the WANCY region and at the 3’ end of the CR, which both contain O_L_-like sequences. The duplication process would occur when both O_L_-like structures are functional. During a mitochondrial replication, after the replication fork arising from the initiation of H-strand synthesis (Figure 2A) passed the WANCY region, where the typical O_L_ is located in most vertebrates, the parental H-strand in this region was exposed as a single strand, and the WANCY tRNA sequence formed an O_L_-like structure (O_L_-like structure 1 in Figure 2B). This structure then initiated an L-strand DNA synthesis event at this site (Nascent L-strand 1 in Figure 2B). Coincidentally, when the replication fork continued to expand and passed the CR, the other O_L_-like sequence was exposed and also formed a O_L_-like structure (O_L_-like structure 2 in Figure 2C), which then initiated a second round of L-strand DNA synthesis (Nascent L-strand 2 in Figure 2C). If both L-strand DNA synthesis events terminated at the WANCY region, the normal termination site, the fragment between the two O_L_-like sequences would be synthesized twice (Figure 2D). By a circular closure event or mitochondrial repair, the 5’ end of Nascent L-strand 1 would connect to the 3’ end of Nascent L-strand 2, while the 3’ end of Nascent L-strand 1 would connect to the 5’ end of Nascent L-strand 2 (Figure 2E). In the next round of mitochondrial replication, the duplication would be made permanent (Figure 2F).

### Speculated TDRL process

The process of tandem duplication followed by random loss discussed above is typical of the TDRL model.

**Figure 3:**
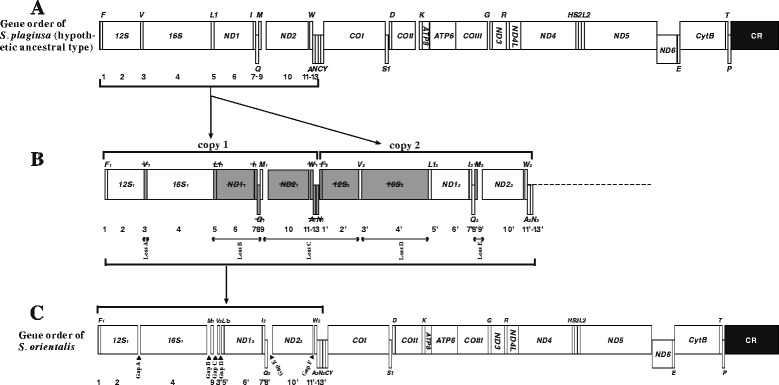
Inferred intermediate steps between the *S. plagiusa* mitogenome (typical ancestral gene order) and the *S. orientalis* mitogenome. Protein-coding genes and CRs are indicated with boxes, and tRNA genes are indicated with columns. Genes labeled above the diagram are encoded by the H-strand and those below the diagram by the L-strand. **(A)** Gene order in the *S. plagiusa* mitogenome; **(B)** the genes between CR and *N* were duplicated and then randomly lost; **(C)** gene order in the *S. orientalis* mitogenome.

Therefore, we applied the TDRL model to describe the rearrangement events that altered the typical gene order (as found in *S. plagiusa*) to that observed in the *S. orientalis* mitogenome. The hypothesized intermediate steps are as follows. First, the above-mentioned double O_L_-like structures initiated DNA synthesis twice during mitochondrial replication, causing tandem duplication of the genes located between the CR and the WANCY region (1–13) (Figure 3 A, B) in the ancestral mitogenome (Figure 3A: typical gene order). In this case, the mitogenome would then have contained two sets of the same gene cluster (Figure 3B: 1–13 and 1’–13’). Because the mitogenome only maintains one set of functional genes, during subsequent evolutionary events, one of each of the 13 duplicated gene pairs randomly lost its function and became a pseudogene (Figure 3B; gray boxes). These pseudogenes then accumulated additional mutations to become shorter non-coding sequences or even be lost from the genome. Eventually, the existing gene order of the *S. orientalis* mitogenome was established (Figure 3C).

### Intergenic spacers provide evidence supporting the model

In the speculated process, after the five duplicated genes or gene clusters lost their functions, as shown in Figure 3B, they would have degraded to form five successive pseudogene fragments or shorter intergenic spacers (Figure 3B, gray boxes). In general, intergenic spacers vanish quickly because the degradation rate of non-functional genes is high to maintain the parsimony of mitogenomes. Interestingly, the speculated intergenic spacers did not disappear, and the positions of the five degraded gene fragments occur in one-to-one correspondence with those of the five residual intergenic spacers in the *S. orientalis* mitogenome: loss A in Figure 3B between *12S* and *16S*, which corresponds to Gap A in Figure 3C; loss B between *16S* and *tRNA-M*, which corresponds to Gap B; loss C between *tRNA-M* and *tRNA-V*, which corresponds to Gap C; loss D between *tRNA-V* and *tRNA-L1*, which corresponds to Gap D; and loss E between *tRNA-Q* and *ND2*, which corresponds to Gap E. The one-to-one correspondence between the loss-of-function fragments and the residual intergenic spacers offers strong evidence supporting the speculated steps of gene duplication and loss in our model (Figure 3).

## Conclusions

In summary, we determined the complete mitochondrial genomes of two *Symphurus* fishes, *S. plagiusa* and *S. orientalis*. The gene order of the *S. plagiusa* mitogenome is the same as that of a typical vertebrate, while that of *S. orientalis* features large-scale gene rearrangements. In the rearranged fragment from the CR to WANCY in the *S. orientalis* mitogenome, six large intergenic spacers more than 20 bp in length remain. The positions of these intergenic spacers occur at a one-to-one correspondence with the loss-of-function fragments in our speculated gene rearrangement model based on TDRL, providing strong evidence for both our model and the TDRL.

The two *Symphurus* fish mitogenomes share another special characteristic: they lack the typical O_L_ region that is conserved in most vertebrates. In addition, in the *S. plagiusa* mitogenome, there are two regions with the potential ability to form O_L_-like structures, which means that both could initiate replication of the L-strand. Accordingly, we speculate that the ancestral mitogenome also possessed these two O_L_-like structures and that during an ancient mitochondrial replication event, both O_L_-like structures initiated DNA synthesis and induced the doubled replication of the L-strand, leading to the duplication of the region between the two structures. The findings of this study are based only on the primary sequence structures of mitogenomes. To substantiate the speculations presented herein, it will be necessary to observe replication intermediates via electron microscopy or 2D-gel analysis.

### Data accessibility

DNA sequences: GenBank accessions JQ639061 and KP992899.

## Additional files

Additional file 1: Table S1. The primers used for fragment amplification of the *Symphurus plagiusa* mitogenome. Additional file 2: Table S2. The primers used for fragment amplification of the *Symphurus orientalis* mitogenome.

## Abbreviations

CR: Control region
WANCY: tRNA cluster of *tRNA-W*, *tRNA-A*, *tRNA-N*, *tRNA-C* and *tRNA-Y*
rRNA: Ribosomal ribonucleic acid
O_L_: Origin for light-strand replication
tRNA: Transfer ribonucleic acid
Mitogenome: mitochondrial genome
TDRL: Tandem Duplication and Random Loss
TDNL: Tandem Duplication and Non-random Loss
DMNR: Dimer-Mitogenome and Non-Random Loss
mtDNA: Mitochondrial DNA
PCR: Polymerase chain reaction
H-strand: Heavy strand
L-strand: Light strand
