# Supplementary material for: Tandem Duplication and Random Loss for mitogenome rearrangement in Symphurus (Teleost: Pleuronectiformes)
Source: BMC Genomics. 2015 May 6;16(1):355. doi: 10.1186/s12864-015-1581-6 (PMC4430869; doi:10.1186/s12864-015-1581-6)
Supplement: Additional file 1: Table S1. — The primers used for fragment amplification of the Symphurus plagiusa mitogenome. [file 12864_2015_1581_MOESM1_ESM.docx]

Table S1. PCR primers for amplification of the fragments in *Symphurus plagiusa* mtDNA

| Forward  primer | Sequence (5’-3’ ) | Reverse  primer | Sequence (5’-3’ ) |
| --- | --- | --- | --- |
| Z15 | ATTAAAGCATAACHCTGAAGATGTTAAGAT | F2671 | AGATAGAAACTGACCTGGAT |
| Z2733 | ATCCAGGTCAGTTTCTATC | F5196 | CTAAATGGTTGGGGTATGG |
| Z2625 | GTTTACGACCTCGATGTTGGATCAGGACAT | F6746 | GCGGTGGATTGTAGACCCATARACAGAGGT |
| R6754 | CTAAGCCATCCTACCTGTG | F11089 | TTTAACCAAGACCRGGTGATTGGAAGTC |
| Z10818 | TTYGAAGCAGCCGCMTGATACTGACAYTT | F12824 | GCAAGAATAAACCATCATCC |
| Z12720 | ATYGCHTATTCATCAGTHGG | F14170 | GTCADGGGTGTAGNCCAAAT |
| Z14170 | ATTCCTCCTCTTTGTGGG | H15149 | AACTGCAGCCCCTCAGAATGATATTTGTCCTCA |
| L14734 | AACCACCGTTGTTATTCAACT | F95 | GACAGTAAAGTCAGGACCAAGCCTTTGTGC |
| L17114 | RCGCCCAAAGCTAGDATTC | F2103 | TTGGGTCCACGATTAGGT |
|  |  |  |  |
