# Supplementary material for: Tandem Duplication and Random Loss for mitogenome rearrangement in Symphurus (Teleost: Pleuronectiformes)
Source: BMC Genomics. 2015 May 6;16(1):355. doi: 10.1186/s12864-015-1581-6 (PMC4430869; doi:10.1186/s12864-015-1581-6)
Supplement: Additional file 2: Table S2. — The primers used for fragment amplification of the Symphurus orientalis mitogenome. [file 12864_2015_1581_MOESM2_ESM.docx]

Table S2. PCR primers for amplification of the fragments in *Symphurus orientalis* mtDNA

| Forward  primer | Sequence (5’-3’ ) | Reverse  primer | Sequence (5’-3’ ) |
| --- | --- | --- | --- |
| Z76 | TTGGTCCTGACTTTACTATC | F2671 | AGATAGAAACTGACCTGGAT |
| Z2625 | GTTTACGACCTCGATGTTGGATCAGGACAT | F4960 | AGTCCCGCCAATGTAAGT |
| Z2733 | ATCCAGGTCAGTTTCTATC | F6746 | GCGGTGGATTGTAGACCCATARACAGAGGT |
| Z6116 | CCCTCCTTACTGCTTCTA | F7258 | GAAAGTTGGGTTGTAGGG |
| Z6296 | CAGACCGCAACCTAAACA | F9851 | AGTTGATAACCCTGAAGTA |
| Z10818 | TTYGAAGCAGCCGCMTGATACTGACAYTT | F13413 | TAGCTGCTACTCGGATTTGCACCAAGAGT |
| Z10389 | TTGCTCATTACCCTCCAC | H15149 | AAACTGCAGCCCCTCAGAATGATATTTGTCCTCA |
| L14734 | AACCACCGTTGTTATTCAACT | F17147 | TAGTTTARTGCGAGAATCCTAGCTTTGGG |
| Z17054 | GYCGGTGGTTARAATCCTCCCTACTGCT | F95 | GACAGTAAAGTCAGGACCAAGCCTTTGTGC |
|  |  |  |  |
